# Supplementary material for: Nafion Swelling in Salt Solutions in a Finite Sized Cell: Curious Phenomena Dependent on Sample Preparation Protocol
Source: Polymers (Basel). 2022 Apr 8;14(8):1511. doi: 10.3390/polym14081511 (PMC9027590; doi:10.3390/polym14081511)
Supplement: Supplementary file 1 [file polymers-14-01511-s001.zip › polymers-1607380-supplementary.pdf]

Nafion Swelling in Salt Solutions in a Finite Sized Cell: Curious Phenomena Dependent on Sample Preparation Protocol

Barry W. Ninham [1], Polina N. Bolotskova [2,3], Sergey V. Gudkov [3], Ekaterina N. Baranova [4,5], Valeriy A. Kozlov [2,3], Alexey V. Shkirin [3,6], Minh Tuan Vu [2], Nikolai F. Bunkin [2,3,*]

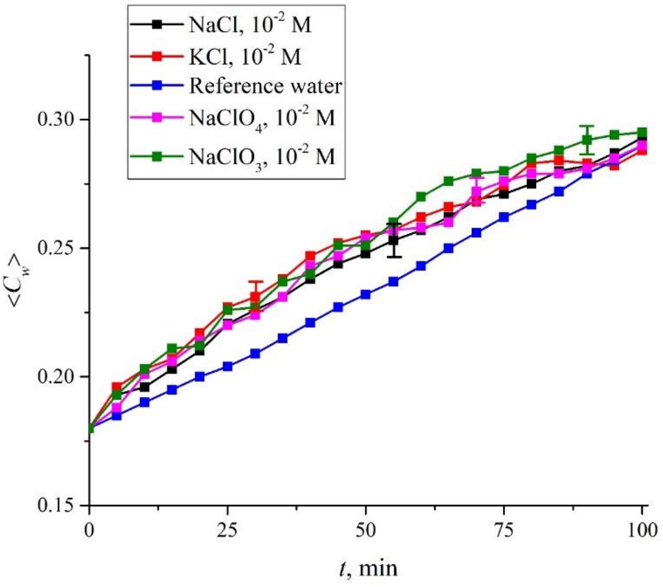

Figure S1. Dependence of the average concentration $\langle C_w(t) \rangle$ for NaCl, KCl, $NaClO_4$ and $NaClO_3$ solutions; the dependence $\langle C_w(t) \rangle$ for deionized (reference) water is also shown. The salt concentration is 10^{-2} M.

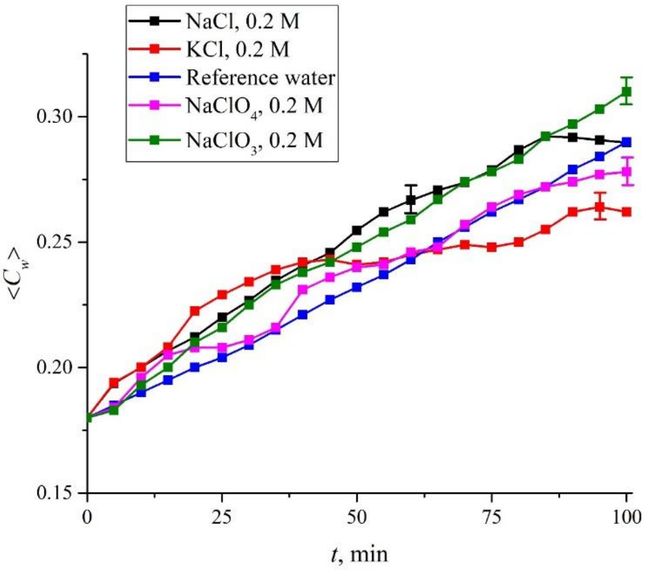

Figure S2. Dependence of the average concentration $\langle C_w(t) \rangle$ for NaCl, KCl, $NaClO_4$ and $NaClO_3$ solutions; the dependence $\langle C_w(t) \rangle$ for deionized (reference) water is also shown. The salt concentration is 0.2 M.

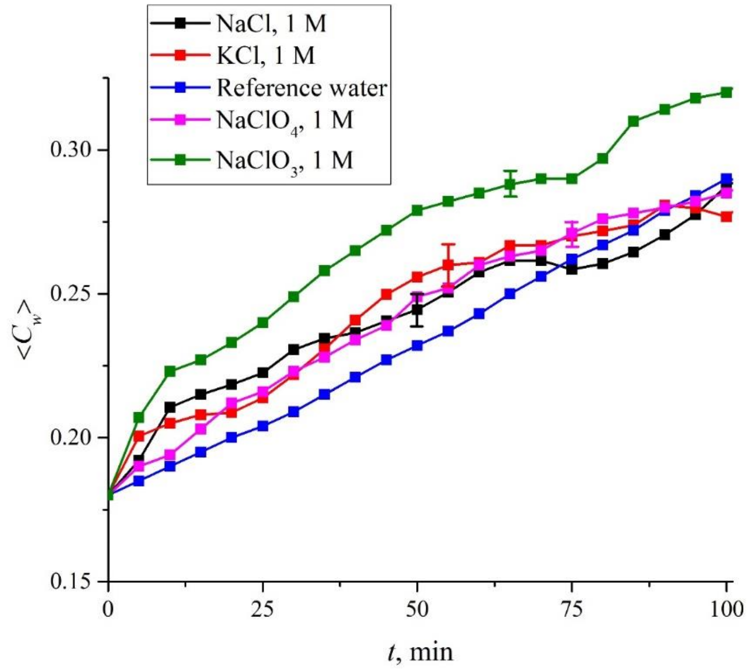

Figure S3. Dependence of the average concentration $\langle C_w(t)\rangle$ for NaCl, KCl, $NaClO_4$ and $NaClO_3$ solutions; the dependence $\langle C_w(t)\rangle$ for deionized (reference) water is also shown. The salt concentration is 1 M.
